# Targeting H19, an Imprinted Long Non-Coding RNA, in Hepatic Functions and Liver Diseases

**DOI:** 10.3390/diseases5010011

**Published:** 2017-03-08

**Authors:** Chad Pope, Shashank Mishra, Joshua Russell, Qingqing Zhou, Xiao-Bo Zhong

**Affiliations:** 1 Department of Pharmaceutical Sciences, School of Pharmacy, University of Connecticut, 69 N Eagleville Road, Storrs, CT 06269, USA; joshua.russell@uconn.edu (J.R.); qingqing.zhou@uconn.edu (Q.Z.); 2 Department of Physiology and Neurobiology, University of Connecticut, 75 N Eagleville Road, Storrs, CT 06269, USA; shashank.mishra@uconn.edu

**Keywords:** H19, liver functions, long non-coding RNA, liver diseases, epigenetics

## Abstract

H19 is a long non-coding RNA regulated by genomic imprinting through methylation at the locus between *H19* and *IGF2*. H19 is important in normal liver development, controlling proliferation and impacting genes involved in an important network controlling fetal development. H19 also plays a major role in disease progression, particularly in hepatocellular carcinoma. H19 participates in the epigenetic regulation of many processes impacting diseases, such as activating the miR-200 pathway by histone acetylation to inhibit the epithelial-mesenchymal transition to suppress tumor metastasis. Furthermore, H19’s normal regulation is disturbed in diseases, such as hepatocellular carcinoma. In this disease, aberrant epigenetic maintenance results in biallelic expression of IGF2, leading to uncontrolled cellular proliferation. This review aims to further research utilizing H19 for drug discovery and the treatment of liver diseases by focusing on both the epigenetic regulation of H19 and how H19 regulates normal liver functions and diseases, particularly by epigenetic mechanisms.

## 1. Introduction

H19, a long non-coding RNA (lncRNA), is both epigenetically regulated and utilizes epigenetic mechanisms to regulate liver cell functions. This review will first describe the history of H19 and then focus on the regulation of the gene expression of H19. It is uniquely expressed from one allele by an intricate process called genomic imprinting. Then, we will examine H19’s roles and implications in various normal functions in liver development and growth, including the regulation of bile acid homeostasis and xenobiotic metabolism. We will discuss dysregulation of H19 in the progression of liver-related diseases, including steatosis, fibrosis, cirrhosis, diabetes, and hepatocellular carcinoma (HCC) with a particular focus on the epithelial to mesenchymal transition (EMT). Finally, we will explore using H19 in therapies for liver diseases either by targeting H19 or by directly using the H19 promoter to drive selective toxicity in cancer. The mechanisms of regulation will be highlighted, emphasizing epigenetic mechanisms.

The discovery and characterization of H19, one of the first lncRNAs described, overviews how lncRNAs were first discovered and assumed to have functions despite not coding for protein. In 1984, the Tilghman lab discovered a RNA transcript that was highly expressed in fetal mouse liver, but decreased in adult liver. They screened a fetal liver cDNA library for moderately abundant clones that hybridized only to a fetal liver cDNA probe, but did not hybridize to an adult liver cDNA probe and other controls; the clone was designated H19 based on its position being the 19th clone in row H. In the discussion of their paper, Pachnis et al. state, “The identity of this protein encoded by H19, if indeed one exists, is unknown at this time” [1]. Further characterization by sequencing revealed that H19 had multiple translation termination signals in all three reading frames, but conversely, was highly conserved. H19 was still transcribed by RNA polymerase II, spliced, and polyadenylated, but puzzlingly did not associate with ribosomes. This dual nature of being similar to protein-coding genes, but incapable of translation led the authors to conclude “…the product of this unusual gene may be an RNA molecule“ [2]. Since then, genome-wide technologies, such as microarrays, particularly tiling arrays, which allow for characterization of sequenced regions where function was not known, resulted in a boom of lncRNA discovery. Technology continues to develop at an alarming rate, and the fascinating H19 discovery was only the beginning of a new field in science.

There are numerous reviews discussing the vast amount of research on H19. Prior reviews cover topics as extensive as H19’s regulation [3,4,5,6] or its broad role in cancer [7,8,9]. Other reviews are more specific to particular types of cancer [10,11,12]. There is a need for more reviews discussing diseases in a particular organ, especially the liver. When H19 is discussed in the context of HCC, reviews examine other lncRNAs in their analysis without focus on H19. This review will describe H19’s role in liver disease, including HCC, with a particular emphasis in any epigenetic regulation where H19 participates. This review will also highlight research from other organ systems that need translational examination in the liver. Overall, we aim to provide a resource for future research on H19, so liver diseases may be treated more effectively in the future.

## 2. Characterization of H19 and Its Participation in Epigenetic Regulation

### 2.1. LncRNAs

LncRNAs are transcripts greater than 200 nucleotides that do not code for proteins. Despite not being translated, approximately 35,000 lncRNAs have been discovered that exhibit characteristics of mRNA, such as being 5′ capped, spliced, and poly-adenylated [13]. Furthermore, lncRNAs are generated similarly to proteins, having similar histone-modification profiles, splicing signals, and exon and intron lengths [14]. LncRNAs are abundant, comprising 80% of all transcripts [15] and their expression levels are highly tissue specific [14].

Molecular functions of lncRNAs are described as signals, decoys, guides, and scaffolds [16]. LncRNAs act effectively as signals due to their specific expression in specific cell types and stages of development as well as their capability to respond to stimuli. Many lncRNAs found in liver currently described in the literature can be classified as signals due to being tissue specific, disease state specific, and/or developmentally specifically expressed. LncRNAs also act as decoys by binding and titrating away proteins. They can be guides capable of binding regulatory molecules, including chromatin remodelers and transcription factors, directing them to specific DNA targets to control gene expression. With different domains capable of binding different molecules, they can also be scaffolds, assembling a complex arrangement of components [16]. Thus, lncRNAs can exhibit many functions and work within the cell to regulate different processes by various molecular mechanisms.

### 2.2. H19

The characteristics of H19 are similar to other lncRNAs in both structure and their temporal and tissue-specific expression pattern. Structurally, the *H19* gene contains five exons and four introns, producing a 2.3-kb lncRNA after splicing. The *H19* gene contains shorter introns than most lncRNA genes, each less than 100 base pairs [17]. It is transcribed from chromosome 7 in the mouse and chromosome 11 in the human. It is adjacent to the protein-coding gene, *insulin-like growth factor 2* (*IGF2*), an important fetal growth factor. These two genes share regulatory sequences required for their expression, including two enhancers located 3’ downstream of *H19* [18]. Their expression is also controlled by an *imprinting control region* (*ICR*) between the two gene loci and exhibits differential methylation [19]. Temporally, H19 is expressed in the embryo, but subsequently downregulated in all tissues, excluding skeletal muscle, shortly after birth [20]. During the fetal and postnatal ages, H19 is abundant in the liver. H19 expression has been shown to correlate with the expression of IGF2, implicating H19’s important role in liver development.

H19 has many diverse biological functions. It is known to participate in the regulation of cell proliferation [21] and differentiation [22], as well as its role in cancer both as an oncogene [23,24] and as a tumor suppressor [25]. In the developing fetus, H19 regulates a number of important genes within the imprinted gene network (IGN), including *Igf2*, responsible for proper embryonic development [26]. Due to participating in many known biological functions and being one of the first lncRNAs discovered and characterized, H19 is one of the most well-studied lncRNAs.

**Figure 1:**
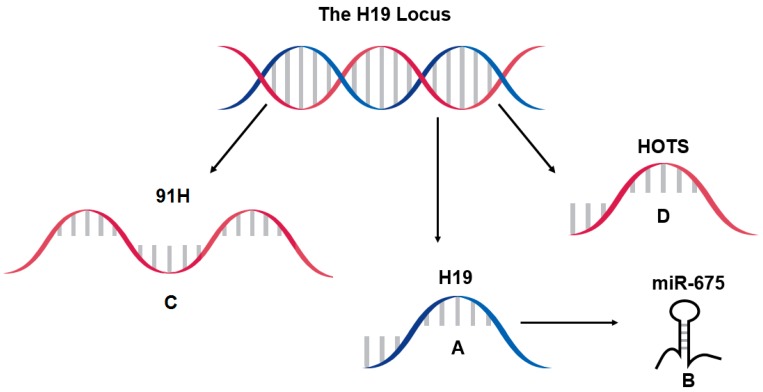
Transcription at the *H19* locus consists of the well-characterized H19 lncRNA (**A**) and its microRNA miR-675 (**B**) encoded within the first exon of *H19*. Two antisense transcripts are also formed from the locus. 91H RNA (**C**) is described as varying in length, but can potentially be encoded from the other DNA strand entirely encompassing *H19* and other portions of its regulatory sequences. HOTS RNA (**D**) is transcribed from most of the antisense sequence of *H19* and upstream bases. HOTS can be translated to form a nucleolar protein.

The *H19* locus harbors multiple transcripts (Figure 1). The main transcript, H19, functions as the full lncRNA, and it encodes within its first exon two variants of microRNA, miR-675 [22]. There are also two antisense transcripts at the locus, *91H* and *HOTS*. The HOTS transcript can produce a nucleolar protein [27], while 91H appears to function solely as an lncRNA [28]. A review and discussion on the antisense transcripts is presented in Section 6.

### 2.3. Regulation of H19 Expression by Epigenetic Mechanisms

H19 is regulated by the epigenetic phenomenon, genomic imprinting. Normally, H19 is only expressed from one parental allele and silenced epigenetically on the reciprocal chromosome. The paternal allele is imprinted, or silenced, due to the highly methylated *ICR* found between *H19* and *IGF2*. The *ICR* is a differentially methylated region (*DMR*) due to having different methylation statuses depending on the chromosomal parental origin. At the maternal allele, H19 is expressed, and on this chromosome, the *ICR* is hypomethylated [29]. *IGF2* is also imprinted, but opposite of *H19*, expression is driven from the paternal allele and the maternal allele is silenced [30]. The same *ICR* also controls *IGF2* imprinting. Located approximately 2 kb upstream of the *H19* promoter between *H19* and *IGF2*, this region regulates monoallelic expression of those genes by being differentially methylated depending on the parental origin of the allele. In normal conditions, the *ICR* is hypermethylated at the paternal allele and hypomethylated at the maternal allele. This balance is important in controlling normal expression of these genes. Deleting 10 kb upstream *H19* disrupts this region and causes biallelic IGF2 expression, thereby disrupting imprinting [31]. The mechanism controlling imprinting of *H19* and *IGF2* involves the binding of either MeCP2 or CTCF based on the methylation status of the *ICR*.

On the hypermethylated *ICR* found on the paternal chromosome, H19 is repressed and IGF2 expressed. H19 expression is silenced through histone deacetylation by MeCP2 binding to methylated CpG dinucleotides. After binding, histone deacetylases (HDACs) interact with MeCP2 to repress H19 [19]. Furthermore, in this state of *ICR* methylation, the *ICR* binds another *DMR* located within *IGF2*, *DMR2*. This *ICR* and *DMR2* interaction moves IGF2 into position to interact with the enhancer region driving IGF2 expression [32]. The binding of the insulator CTCF to the *ICR* is disallowed due to the high number of methyl groups [32].

Conversely, on the hypomethylated *ICR* found on the maternal chromosome, H19 expression is not repressed by MeCP2 binding and able to interact with its enhancer region. CTCF is allowed to bind, insulating and preventing the expression of IGF2 by two known mechanism. First, CTCF binding allows the interaction of the *ICR* with *matrix attachment region 3* (*MAR3*) and another *DMR*, *DMR1*, which is located in an intergenic region of *IGF2*. These interactions result in a loop around *IGF2* and impediment of expression [32], as well as disallowing the enhancers from interacting with *IGF2* [33]. Specifically in liver, the binding of CTCF to the *ICR* is mediated by the interaction of CTCF with prohibitin 1 [34]. Second, CTCF interacts with the *IGF2* promoter region and recruits polycomb repressive complexes leading to H3K27 methylation causing suppression of IGF2 [35].

In summary, two states on separate parental alleles exist in normal conditions when H19 and IGF2 are co-expressed. On the paternal allele, the *ICR* is methylated, and the enhancer region is able to interact with *IGF2* to allow expression. On the maternal allele, the *ICR* is not methylated, allowing the same enhancer region to, instead, drive H19 expression. Therefore, this region and its methylation status are critical for both H19 and IGF2 expression, expressed coordinately albeit from opposite alleles.

### 2.4. H19 Regulates Gene Expression by Epigenetic Mechanisms

Activating or repressing H19 may be important as an epigenetic therapy for future treatment of epigenetic abnormalities in diseases. In this section, we aim to highlight known cases where H19 directly participates in either the repression or activation of transcription through direct epigenetic mechanisms. First, we will examine H19 in the repression of genes and, second, in the activation of genes. Finally, we will examine how H19’s role as an epigenetic modifier impacts chemotherapy resistance. In these examples, H19 plays a clear role in affecting transcription through chromatin remodeling.

**Figure 2:**
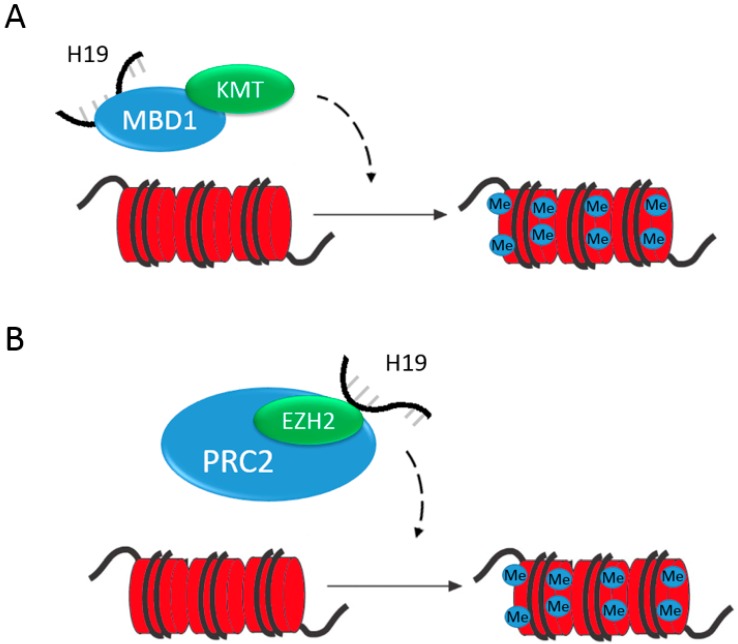
Epigenetic regulation by H19. H19 acts to repress genes within the imprinted gene network, such as *IGF2*. H19 binds MBD1. MBD1 then binds methylated DNA and then recruits histone lysine methyltransferases (KMTs) to silence these genes by chromatin compaction (**A**). H19 silences E-cadherin. H19 binds EZH2, a H3K27 methyltransferase part of the PRC2, causing downregulation of *E-cadherin* (**B**).

In many examples, H19 recruits epigenetic modifiers, acting as a guide to repress gene expression. Important for embryonic development, H19 controls at least nine imprinted genes (*Igf2*, *Igf1r*, *Dlk1*, *Meg3*, *Slc38a4*, *Peg1*, *Dcn*, *Cdkn1c* and *Gnas*) in the mouse IGN [36]. The control over some of these genes occurs by H19 interaction with methyl-CpG-binding domain protein 1 (MBD1), a protein in the same family as MeCP2 (discussed earlier as a repressor of H19 expression). MBD1 binds to methylated DNA to recruit HDACs and histone lysine methyltransferase (KMT)-containing complexes, SETDB1 and SUV39H1, silencing genes via H3K9 methylation, inducing chromatin compaction (Figure 2A) [36]. In a second example, H19 represses gene expression through the interaction with enhancer of zeste homolog 2 (EZH2), a H3K27 methyltransferase in a part of the polycomb repressive complex 2 (PRC2) in bladder cancer. H19 association with EZH2 results in the activation in Wnt signaling and recruitment of PRC2 to silence E-cadherin (Figure 2B) [37]. Although, the direct H19 and EZH2 binding has not been determined in liver, EZH2 has been shown to silence tumor suppressor microRNAs in liver cancer and is upregulated in HCC [38]. It would be interesting to examine H19’s role in HCC to determine if they are binding partners in this condition.

**Figure 3:**
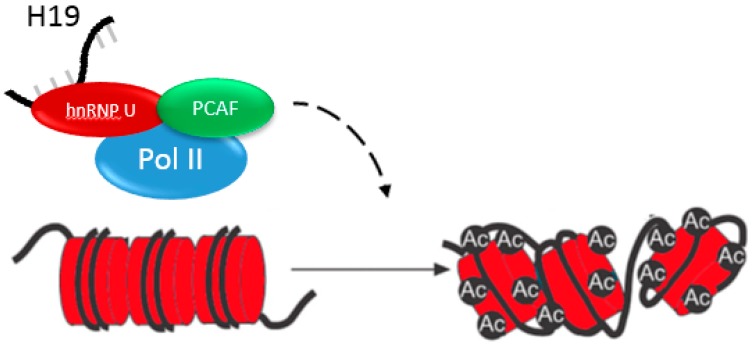
H19 can also activate gene transcription. H19 binds hnRNP U, which is a member of the complex with RNA Polymerase II and PCAF, a histone acetyltransferase. H19 binding results in the upregulation of the miR-200 family of microRNAs through histone H3 acetylation.

H19 can also promote gene expression by acting as a guide for epigenetic modifying enzymes. For example, H19 binds hnRNP U, part of a complex with RNA polymerase II and a histone acetyltransferase, PCAF. Histone acetylation results in the upregulation of genes within the miR-200 family. Activation of the miR-200 family ultimately suppresses metastasis in HCC (Figure 3) [39]. These examples highlight how H19 can both repress and activate gene expression by epigenetic mechanisms, making context crucial in understanding its functions.

H19’s role as an epigenetic modifier may also be important in the study of a barrier in cancer treatment, chemotherapy drug resistance. P-glycoprotein, an efflux transporter, is often upregulated in cancer cells. This prevents cancer drugs from accumulating in cancer cells, thereby reducing their efficacy. H19 may induce P-glycoprotein expression by regulating *MDR1* promoter methylation [40]. In a doxorubicin-resistant hepatocellular carcinoma cell line, R-HepG2, it was found that knockdown of H19 resulted in an increase of promoter methylation at the *MDR1* promoter. Establishment of the mechanism, by which H19 regulates the promoter methylation status of efflux transporters in chemotherapy drug resistance, will further define H19’s role to influence gene expression as an epigenetic modifier.

## 3. Characterization of H19 and Its Participation in Epigenetic Regulation

### 3.1. The Roles of H19 in Liver Development

The liver is an essential organ for life. Hepatocytes, the main parenchyma of the liver, comprise almost 80% of the adult liver’s total mass [41]. The remaining mass includes cholangiocyte parenchyma and the non-parenchymal cells, including sinusoidal endothelial, stromal, Kupffer, and stellate cells. The liver has many important biological functions, such as detoxification, modification and excretion of exogenous and endogenous substances, the synthesis of cholesterol, bile salts, and phospholipids, and blood glucose regulation [42]. The liver is also the major organ for hematopoiesis in the fetus [43]. We will examine H19’s role in some of these major processes, including its role when these processes are not functioning normally in disease.

Many studies have shown that lncRNAs are important regulators of normal liver development. LncRNAs are differentially expressed throughout different stages of development from embryogenesis to adult life. Our lab has previously characterized, using whole transcriptome analysis, the expression patterns of liver lncRNAs by RNA-sequencing in mice before birth to adult [44]. We have discovered that there are three major oncogenic patterns of differential expression and potentially a functional transition of lncRNAs that occur at the neonatal, adolescent, and adult stages, implying their importance in liver maturation. H19 was found to be the most differentially expressed annotated lncRNA between fetal and adult liver. Mechanistically, H19’s drastic silencing of expression at the postnatal age is attributed to transcriptional repression by zinc fingers and homeoboxes 2 (Zhx2) protein [45], the same protein that regulates alpha-fetoprotein, which led to H19’s initial discovery [1]. This intricate temporal control indicates H19 is a regulator of liver development.

H19 controls liver growth and potentially the decline of the hematopoietic role of the liver after birth through the IGN. The decline of the expression of genes within the IGN coordinates the deceleration of organ growth, including the liver after birth [46]. As previously described, H19 has been shown to regulate genes within the IGN by epigenetic mechanisms [36], indicating that H19 has a major influence on development. Igf2 and Dlk1, both in the IGN, are also important for hematopoiesis [47,48], and the liver is the main site of hematopoiesis during birth. The repression of Igf2 and Dlk1 by H19, which has been shown experimentally, indicates that H19 is potentially responsible for the developmental switch of the liver starting as a hematopoietic organ to an organ that focuses mainly in metabolism. H19’s control over the IGN leads to many implications on its role in regulating liver development.

H19 also regulates the proliferation of liver in development by Wnt signaling. The Wnt signaling pathway is critical for postnatal liver growth [49]. H19 inhibits proliferation in fetal liver by inhibiting the canonical Wnt signaling pathway through inhibition of its major intracellular signal transducer, β-catenin [50]. H19 can block the interaction of hnRNP U with actin, leading to transcriptional repression of genes involved in the Wnt signaling pathway [50]. Despite these two mechanisms, there is also evidence of H19 activating Wnt signaling, albeit in a different context and cell type: bladder cancer [37]. These examples show how H19 can act by different mechanisms to affect one master regulation pathway. In the context of liver proliferation in development, H19 appears to be a negative regulator of Wnt signaling.

The microRNA encoded within *H19*, miR-675, is also potentially important for development. The RNA binding protein, HuR, blocks the processing of miR-675 from *H19*, but during gestation, HuR is downregulated, causing miR-675 expression. Overexpression of miR-675 in extraembryonic cell lines causes reduced proliferation. Mechanistically, miR-675 inhibits Igf1r, and this inhibition can limit placental growth [51].

H19 is also implicated in abnormal fetal development, regulated by epigenetic mechanisms. Developmental-specific methylation occurs at different regions around the *H19/IGF2* locus in *DMRs* in promoters for both genes [52]. DNA methylation of imprinted genes is first erased in primordial germ cells and reestablished later in the formation of gametes [53]. *In vitro* fertilization (IVF) may disrupt normal embryonic and fetal growth, causing abnormal gene expression in liver. Through disruption of epigenetic regulatory networks, errors on both the maternal and paternal alleles occur at the *H19 DMR* and the *IGF2 DMR2*, respectively. H19 is significantly downregulated, and IGF2 is upregulated in livers of mice at birth and three weeks of age if they were conceived via IVF. At 1.5 years of age, mice conceived via IVF had significantly lower H19 and IGF2 expression in liver. IVF causes hypomethylation at the *H19 DMR*, indicating that early life manipulation affects vulnerability to differential methylation. In humans, growth disorders are higher and birth weights are lower in newborns conceived by IVF [54]. This study highlights the importance of maintaining proper methylation statuses at the *H19/IGF2* locus in development.

### 3.2. The Roles of H19 in the Regulation of Xenobiotic Metabolism and Transport

The liver is the most important organ for metabolizing endogenous compounds and xenobiotics, including drugs. The liver contains multiple classes and families of metabolizing enzymes. These enzymes participate in reactions to make xenobiotic compounds more water soluble and capable of clearance through excretion in the urine or transportation into the bile. For example, the cytochrome P450 enzymes are a class of phase I enzymes. They typically function as monooxygenases that insert oxygen into molecules, making them more water soluble and easier to clear from the body [55]. Phase II enzymes are drug-metabolizing enzymes capable of conjugation reactions [56]. Drug transporters are also important for clearing xenobiotics from the body [57]. Efflux transporters, such as MDR1, are upregulated in HCC [58], often inhibiting chemotherapy drugs from effectively inhibiting cancer cells. Currently, there is little research on how lncRNAs affect xenobiotic metabolizing enzymes, particularly in mammalian systems, indicating a knowledge gap and a potential area for further study.

H19 affects drug transporter and phase II conjugation, resulting in alteration of the metabolism and disposition of drugs. H19 is overexpressed in a number of drug-resistant cell lines, including doxorubicin-resistant liver cancer cells [40] and cisplatin-resistant ovarian cancer A2780-DR cells [59]. In both papers, knockdown of H19 expression results in restored chemotherapeutic sensitivity. The doxorubicin-resistant liver cancer cells exhibit an overexpression of the transporter MDR1. Sensitivity is restored by methylation of the *MDR1* promoter, causing its repression and inability to efflux the drug [40]. In the cisplatin-resistant ovarian cancer cells, H19 knockdown coincided with a reduction of glutathione S-transferase P1 (GSTP1), responsible for cisplatin inactivation [59]. Examination of changes in phase II glutathione conjugation and in drug transporters after H19 knockdown in liver cancer cell lines should further be explored, as these enzymes are important for clearing drugs and for the accumulation of drugs in cancer cells.

Although H19 has not been directly linked to the regulation of phase I metabolism enzymes, current research may support H19 having a role. Gene expression profiles of P450 enzymes change in the developing liver and the normal adult P450 expression is not established in mice until a specific postnatal age [60]. This change corresponds inversely with the expression pattern of H19 in liver. ZHX2, described earlier as the repressor that targets H19 for silencing in postnatal liver, also regulates sexually dimorphic, developmentally-regulated P450 genes in the liver, including *Cyp2a4*, *Cyp2b13* and *Cyp4a12* [61]. Human *CYP2A6* has the highest homology to mouse *Cyp2a4*, and CYP2A6 is responsible for metabolizing nicotine, carcinogens and several pharmaceuticals. Although there is no direct link of H19’s role in regulating these enzymes, its direct repressor has been described as important. Further research is needed to determine if H19 regulates phase I metabolism and its implication on the metabolism of drugs, especially in the fetus and in postnatal liver. This research will aid our understanding of how the fetus, neonates, and children handle drugs while they express H19 compared to adults without H19 expression in the liver.

## 4. The Roles of H19 in the Progression of Liver Diseases

### 4.1. The Roles of H19 in the Development of Steatosis, Fibrosis, and Cirrhosis

Targeting H19 in cholestatic liver fibrosis may reduce liver injury. Cholestasis is caused by the buildup of cytotoxic bile acids due to bile acid synthesis and accumulation from blocked uptake into hepatocytes and inhibited renal excretion via NTCP [62]. The accumulation of bile acid leads to cell injury, causing inflammation and fibrosis [63]. Although H19 does not seem to play a role in bile acid synthesis, its direct repressor, SHP, represses CYP7A1 and CYP8B1 after activation by FXR. The antiapoptotic protein, BCL2, appears to have an overarching control over SHP and H19. Mechanistically, BCL2 overexpression results in a drastic 47-fold increase in H19 due to its degradation of the SHP repressor. The molecular basis, by which H19 regulates liver fibrosis, has yet to be completely determined. Despite not directly controlling P450s or FXR expression, the knockdown of H19 still partially rescues BCL2-induced liver injury [62]. This partial rescue of injury may be an important observation for the treatment of cholestatic liver fibrosis pointing to H19 as a target for potential therapy.

Another condition, non-alcoholic fatty liver disease (NAFLD), occurs when fat is deposited in liver, steatosis, and lncRNAs, including H19 and its co-regulated protein-coding partner, IGF2, may play important roles in this disease progression. One study found that approximately 500 lncRNAs were upregulated and 1200 lncRNAs were downregulated in human livers of patients with NAFLD compared to healthy livers [64]. Another study links H19 to steatosis through PLIN2, a member of the lipid droplet protein family that is markedly increased in fatty liver. When PLIN2 was inhibited, a 548-fold increase in H19 was observed with a significant decrease in liver triglycerides [65]. In another study, p62, a binding protein of IGF2 mRNA, was highly expressed in diseased liver. When overexpressed in mice, p62 can induce the steatotic phenotype [66], including a two-fold increase in triglycerides compared to wildtype [67]. It was first found that p62 expression did not induce inflammation, showing correlation to NAFLD, but not progressing fully to NASH. However, other researchers have reported p62 overexpression resulting in the activation of NF-κB, suggesting an increased inflammatory response and progression to NASH [68].

NAFLD can progress to its most extreme form, non-alcoholic steatohepatitis (NASH). NASH is the major cause of cirrhosis of the liver. Cirrhosis, a disease where the liver does not function properly due to long-term damage, resulted in 1.2 million deaths in 2013 [69]. Cirrhosis is usually caused by alcohol or hepatitis B or C. Fibrosis, or scarring of the liver, and steatosis, fatty liver, often precedes cirrhosis. Hepatic stellate cells are the main extracellular matrix producing cells and upon activation can promote fibrosis and cirrhosis. H19 is known to sequester miR-874 in the intestinal barrier [70], and this microRNA is upregulated in hepatic stellate cells upon activation, potentially implicating a mechanism that needs further study [71]. Furthermore, whole transcriptome RNA-sequencing analysis was performed in cirrhotic livers compared to normal healthy tissue, and H19 was discovered to be upregulated in cirrhotic liver tissue [72]. Only weak correlation of H19 to this disease has been reported, and these studies do not examine mechanism, again indicating a need for further research. It is clear, however, that lncRNAs, including H19, are involved in NAFLD and potentially to its progression to NASH.

### 4.2. The Roles of H19 in the Regulation of Diabetes

The body needs to maintain blood sugar at precise levels and the liver is a major site of glucose regulation. In times of low blood sugar, the alpha cells of the pancreas secrete glucagon, stimulating the liver to release glucose stores and induce the production of more glucose through glycogenolysis. Conversely, glycogenesis, the process of generating glycogen from glucose, is stimulated by insulin generated in the beta cells of the pancreas when blood glucose levels are high. Insulin promotes the liver and muscle to take up blood glucose, effectively dropping blood glucose levels. Diabetes mellitus (DM) is a disease caused by prolonged increased blood sugar levels due to dysregulation of these processes. DM is caused by either the pancreas not producing enough insulin (type I DM) or the body, including the liver, not properly responding to insulin (type II DM) [73]. 

There is superficial evidence for H19’s significance in type II DM. Smooth muscle cells cultured in the presence of insulin express a five-fold increase in H19 than when cultured in media alone [74]. This indicates that H19, at the very least, responds to changes in insulin. Mice with a whole-body knockout of H19 expression (through targeted deletion at the maternal allele) are 27% heavier [75] due to this mutation, which also results in biallelic expression of IGF2 [31] and improper growth after birth, predisposing them to type II DM [76]. These observations begin the story, but more convincing mechanistic studies examining changes in the epigenetics profiles of *H19* and *IGF2* are needed to fully determine H19’s role.

Epigenetic phenomenon in the liver at the *ICR* between *H19* and *IGF2* and H19 expression are proposed to play a role in type II DM. In a study examining differential mRNA expression levels and patterns of DNA methylation in liver tissue, significant differences were observed between normal patients and patients with type II DM, such as an increase in H19 expression and the degree of methylation at its gene locus [77]. Another study reported a significant degree of hypomethylation at the *H19* locus in an insulin-resistant female [78]. Currently, there is little research on the mechanism of insulin resistance in the liver regarding H19; only associations regarding expression and methylation status have been characterized. Because the liver is a major site of insulin resistance in type II DM, there is the need for further research in liver, specifically. There is also significant evidence that H19 is important in other tissues, such as insulin resistance in muscle and insulin production in the pancreas due to glucose intolerance.

H19 plays a role in both humans and mice with type II diabetes in the muscle. H19 was shown to be significantly decreased and to act as a decoy for microRNA let-7 [79]. H19 acts as a sponge that sequesters let-7, which targets genes, such as *Insr* and *Lpl*, to inhibit the insulin-PI3K-mTOR pathway. Downregulation of H19 in diabetic muscle limits let-7 sequestration, subsequently increasing inhibition of the pathway and promoting insulin resistance. Let-7 has also been reported to destabilize and downregulate H19 as a protective mechanism in hyperinsulinemic conditions in non-diabetic muscle cells via a KSRP-dependent, insulin/PI3K/AKT signaling system. Chronic downregulation of H19 limits glycogenesis and interferes with normal glucose metabolism [79]. Examining how H19 interacts with let-7 in liver may yield parallel mechanisms. Let-7 is expressed in liver, and as stated earlier, H19 expression was shown to increase in patients with type II DM. Another example of H19 sequestering microRNAs affecting important signaling pathways important for glucose regulation was studied in tendon-derived stem cells. Here, H19 directly targets miR-29b-3p [80]. This microRNA has also been shown to lead to dysregulation of insulin/PI3K-AKT signaling in both the livers and pancreases in a mouse model that develops hyperglycemia [81]. Translational research is needed to further elucidate H19’s role on these targets in liver.

The pancreas is another site of differential methylation of the *H19 ICR* in mice exposed to high intrauterine glucose, resulting in diabetes. Researchers induced diabetes in pregnant females. As a result, their pups received a high exposure to glucose, leading to low expression levels of H19 and IGF2. Low expression was due to hypermethylation of the *ICR* in the pups’ pancreatic islets. Low expression of H19 and IGF2 was also observed in the sperm of these mice. This indicates that high intrauterine glucose exposure can cause glucose intolerance, and this disease state can be passed down further to future offspring [82]. This study provides a compelling mechanism for the inheritance of childhood diabetes with H19 as a significant factor.

H19 may act differently in different tissues impacting diabetes in a multifaceted fashion. Diabetes linked to H19 in liver has been severely understudied, despite being an important organ in insulin resistance. H19 has been shown to regulate glucose intolerance and insulin resistance, but studies have mainly focused on muscle and the pancreas. Discovering how H19 participates in this disorder in the liver will allow for a more systemic understanding of the problem.

### 4.3. The Roles of H19 in Hepatocellular Carcinoma

Hepatocellular carcinoma (HCC) is a serious disease with few treatment options, needing more targeted therapies for better treatment. HCC is the fifth most common cancer [83] and the most common liver cancer, accounting for 75% of all primary cases [84]. HCC has many known risk factors, including hepatitis B and C [85]. Treatment includes liver transplantation with a survival rate ranging from 67% to 91% from studies performed in the late 2000s [86], pharmacological intervention with a tyrosine kinase inhibitor, sorafenib, for inhibiting tumor-cell proliferation [87] or surgical resection [88]. HCC is the most studied liver disease, and there have been many newly-discovered roles that various lncRNAs play in its progression, paving the way for new drug targets.

H19’s importance has been widely implicated in HCC; however, reports are inconsistent in its role of promoting cancer as an oncogene or acting as a tumor suppressor. Raveh et al. recently published an excellent extensive review regarding H19’s role in cancer initiation, progression and metastasis, where they aimed to resolve conflicting literature [9]. However, there are many differences in HCC compared to other cancers, so the need to concentrate attention on mechanisms specific to liver is important. For instance, there is a difference in how p53, the tumor suppressor regarded as guardian of the genome, behaves in different cancers. It appears p53 does not control the reemergence of H19 expression in HCC as it functions in other cell types. In HeLa cells derived from cervical cancer, repression of H19 by p53 was observed at the *H19* promoter [89]. In the thymus, p53 also suppresses H19 by regulating DNA methyltransferase expression profiles. This causes methylation at the *H19 ICR*, leading to changes in H19 and IGF2 expression [90]. In the liver, however, p53 does not contribute to the methylation status of the *ICR*, and insignificant changes in H19 and IGF2 expression were observed after knockout of p53 [90]. This example highlights how H19 is regulated differently between different cancers and cell types, as well as the need to focus on H19 in the context of HCC.

Many examples discuss H19 promoting cancer. The reemergence of H19 and IGF2 expression in HCC alone implicates them as positive regulators. After H19 and IGF2 are downregulated at the postnatal ages, they are both reactivated to be expressed in adult livers with HCC [91,92,93]. Both genes exhibit biallelic expression due to a dysregulation of their imprinting [93]. H19 re-expression might be explained by repression of Zhx2, the gene that silences H19 at the postnatal age, in HCC through promoter methylation [94]. Targeting H19 expression may be a potential therapy, as H19 knockdown in cancer cell lines, such as Hep3B, has been shown to decrease tumor weight and tumor volume [95].

HCC tumorigenesis promotion is also regulated by miR-675 through various mechanisms. First, miR-675 directly increases proliferation in HCC by affecting cell cycle regulation through the inhibition of retinoblastoma protein [96]. Second, miR-675 upregulates H19 expression. Molecularly, miR-675 inhibits HP1α causing histone modifications (reduced H3K9 trimethylation, reduced H3K27 trimethylation and increased H3K27 acetylation) at the *EGR1* promoter, enhancing its transcription. EGR1, in turn, upregulates H19, activating PKM2. Ultimately, this results in tumor formation and the promotion of angiogenesis [97].

Tumors transitioning from benign to malignant undergo angiogenesis, and H19 impacts this area of tumorigenesis, as well. To stimulate angiogenesis, H19 has been found to be released from the exosomes of CD90+ liver cells to endothelial cells. H19 then induces the expression of various transcripts, such as VEGF, known to stimulate angiogenesis in endothelial cells [98]. Angiogenesis inhibitors are used in the treatment of cancer pointing to H19 as a potential target in this example. Previously mentioned sorafenib displays antiangiogenic activity outside of its main mechanism to suppress tumor growth [99]. Suppressing H19 may also help to treat HCC through inhibition of angiogenesis.

Other lncRNAs regulate liver cancer in tandem with H19. Recent studies have suggested that initiation of HCC can start with progenitor cells of the liver rather than the parenchyma. The lncRNA CUDR accelerates liver cancer stem cell growth by binding cyclinD1 and, as a complex, binding both the promoters of *H19* and *c-Myc*. Increased expression of H19 promotes excessive TERT enhancing telomerase activity, and c-Myc increases liver cancer stem cell proliferation [100]. These well-defined mechanisms support the role of H19 being an oncogene. However, H19 can also assume a role in contrary to the promotion of HCC. There is increasing evidence that supports H19 as being a tumor suppressor acting to inhibit metastasis.

### 4.4. The Roles of H19 in the Epithelial-to-Mesenchymal Transition

Tumor metastasis requires the ability of cancer cells from a primary site to invade a secondary site. In order for this to occur, the cancer must transition from its originating cell type to a cell type capable of differentiating into various other cell types. EMT occurs when epithelial cells become mesenchymal cells with this capability. An original cell loses its polarity and cell-to-cell adhesion, gaining migratory and invasive properties. This gives the cell the ability to differentiate back into a cell type of the tissue it has invaded.

Epigenetic changes are involved in cancer metastasis in HCC and H19 has been shown to impact pathways, resulting in epigenetic changes and leading to tumor suppression. The next example was previously discussed in a prior section examining H19 regulation by epigenetic mechanisms. H19 suppresses metastasis by repressing markers for EMT through the regulation of the miR-200 family. The markers for EMT examined (E-cadherin, cytokeratin-8, cytokeratin-19, and claudin 1) were increased following H19 knockdown, indicating that H19 inhibits EMT. H19 accomplishes this via histone acetylation to activate the miR-200 pathway after complexing with hnRNP U/PCAF/RNAPol II [39]. The miR-200 family was found to enhance E-cadherin expression in two different HCC cell lines [101]. The miR-200 family achieved this enhancement by directly targeting E-cadherin’s transcriptional repressors, ZEB1 and ZEB2, as determined in NMuMG murine mammary epithelial cells [102]. E-cadherin expression enhancement hinders EMT in an *in vitro* model of EMT induced by transforming growth factor-β in NmuMG cells. E-cadherin expression also decreases motility in different cell lines derived from cancer, including HepJ5 cells, an HCC cell line [101]. Again, the H19 transcript is not fully responsible for its function in EMT, as miR-675 also plays a role. Evasiveness is reduced by inhibiting Twist1, a key mediator of EMT [96]. Despite the previous assumption that H19 promotes HCC by increasing proliferation and angiogenesis, H19 opposes metastasis. Understanding this complicated interplay between *H19* acting as an oncogene and a tumor suppressor results in a more complex, but clearer picture of how H19 participates in each role.

## 5. Targeting H19 for Development of Therapeutic Approaches for Liver Diseases

New therapies to treat liver diseases are needed and more research will need to follow before effectively utilizing H19. Currently, there is very little research connecting H19 to diseases, such as type II DM or NAFLD and NASH, and more studies are needed before therapies can be designed utilizing H19 as a target. However, there is exciting research using H19 to treat HCC, which is greatly needed. Currently, there is only one approved pharmacological intervention to treat HCC, sorafenib. In 2008, it was found that survival is extended only three months for patients with advanced HCC after treatment with sorafenib [87]. H19 has been suggested as a candidate tumor marker for HCC [103]. Although diagnosis is an important step to treatment, it is more powerful to think of directly targeting or using *H19* in a gene therapy approach. DTA-H19 is a plasmid containing a diphtheria toxin ‘A’ chain and expression is driven by the *H19* promoter. Toxicity is highly selective, because cancerous cells have been shown to activate the *H19* promoter and normal healthy cells do not. Current research has displayed a delay in tumor growth and tumor regression of colon adenocarcinoma metastases in the livers of rat [104], as well as clinical trials for bladder and ovarian cancer by the company BioCancell (Jerusalem, Israel), (http://www.biocancell.com/lead-program/bc-819/). This indicates the potential for novel gene therapies using lncRNAs, such as H19, promising new drug modalities.

Drug resistance is a major problem in chemotherapy. In the two examples regarding chemotherapy drug metabolism and transport presented earlier, there is promise in targeting H19. Often, MDR1 is overexpressed, leading to increased efflux of cancer drugs and inefficacy. Targeting H19 in cancer therapy may reduce MDR1-associated drug resistance, as H19 knockdown has been shown to suppress MDR1 expression. Suppression is through increasing *MDR1* promoter methylation, leading to increased accumulation and efficacy/toxicity of doxorubicin in doxorubicin-resistant R-HepG2 cells [40]. The cisplatin-resistant cell line A2780-DR cells also become chemosensitive with knockdown of H19 [59]. These two examples are potentially important in the fight against drug resistance and treating cancers.

## 6. Further Considerations at the *H19* Locus, *91H* and *HOTS*

Genes with transcription overlapping *H19* have been discovered other than H19’s well-studied microRNA, miR-675 (Figure 1). *91H* and *H19 opposite tumor suppressor* (*HOTS*) are both antisense to *H19* and have been discussed in the context of disease. Their roles in liver have yet to be determined, leaving the door open for further research. Due to their implication in other diseases, their sharing of sequence, and either their similar regulation with or regulation of H19, it is important to discuss them here.

*91H* in human is a potentially 120 kb long transcript antisense to *H19*. At full-length, it overlaps the entire *H19* gene, the *ICR* between *H19* and *IGF2*, and the previously discussed enhancers that drive expression of H19 and IGF2 [28]. Like H19, 91H is also upregulated in a number of cancers [28,105,106,107] and mirrors studies examining H19’s role as a tumor suppressor and an oncogene with no clear consensus of the overall general mechanism. 91H is also important for the regulation of IGF2 expression. Knockdown studies have shown that 91H contributes to IGF2 expression at the paternal allele [28]. 91H is also responsible for maintaining *H19/IGF2* imprinting and preventing DNA methylation on the *H19/IGF2* locus (*ICR* and *H19* promoter) on the maternal allele, potentially by binding and masking these sites, driving H19 and IGF2 expression [108]. Outside of controlling imprinting, 91H can also directly activate a promoter of *IGF2*. This activation can be counteracted by H19 [109]. In order to develop future targets for therapies, it may be important to understand these mechanisms of 91H that have a direct effect on H19 expression, as well as 91H’s role in normal liver function, including its potential to cause liver disease.

*HOTS* is another gene transcribed within the H19 locus that overlaps all but the first exon, first intron, and most of the second exon of *H19* in human. HOTS is maternally expressed and imprinted like H19, but unlike H19, it codes for a protein. When overexpressed, HOTS inhibits various tumor types, and knockdown of HOTS results in tumor growth. In samples of Wilms tumors, it was observed that a loss of imprinting at the locus that results in biallelic expression of IGF2 also silences HOTS. This led the authors to conclude that HOTS is a tumor suppressor [27]. With little research on this transcript and its protein, it is difficult to assume that it is a useful target in therapy. However, targeted overexpression or gene therapy using HOTS may be useful to suppress cancer. As more information was discerned over the years studying H19, its role in disease was discovered to be immensely complex. The same could be true in further investigations of both 91H and HOTS.

## 7. Conclusions

lncRNA research is a relatively new focus. Since H19 was one of the first lncRNAs to be identified and characterized, there is an abundant amount of research depicting its various roles. The epigenetic regulation of *H19* is unique and complex, being an imprinted gene containing a regulatory region that is differentially methylated depending on the parental origin. H19 participates in normal liver functions, such as development, and its dysregulation occurs in many liver disease, including HCC, type II DM, NAFLD, NASH, and cholestatic liver fibrosis. lncRNAs, including H19, have unique regulatory abilities, capable of binding proteins. They are also capable of participating in epigenetic modification of genes to affect gene expression in numerous signaling pathways or are direct effectors of processes in normal function and disease. Many mechanisms have been presented, indicating that H19 is a complex actor with roles in many organ systems, times of development, and in many disease states. Its complex role in HCC has shown to act as an oncogene regulating proliferation and angiogenesis while unusually also to act as a tumor suppressor inhibiting metastasis. As the science progresses and gives us new insights into how lncRNAs and H19 control normal liver function and disease, therapies to treat disorders will follow eventually to improve overall human health.

## Abbreviations

The following abbreviations are used in the manuscript:

BCL2	B-cell lymphoma 2
CTCF	CCCTC-binding factor
CDKN1C	Cyclin-dependent kinase inhibitor 1C
DCN	Decorin
DLK1	Delta-like non-canonical notch ligand 1
DM	Diabetes mellitus
DMR	Differentially-methylated region
EGR1	Early growth response protein 1
EMT	Epithelial-mesenchymal transition
FXR	Farnesoid X receptor
EZH2	Enhancer of zeste homolog 2
GSTP1	Glutathione S-transferase pi 1
GNAS	GNAS complex locus
H3K9	Histone H3 at lysine 9
H3K27	Histone H3 at lysine 27
HCC	Hepatocellular carcinoma
HDAC	Histone deacetylase
HnRNP U	Heterogeneous nuclear ribonucleoprotein U
HOTS	H19 opposite tumor suppressor
ICR	Imprinting control region
IGF2	Insulin-like growth factor 2
IGF1R	Insulin-like growth factor 1 receptor
IGN	Imprinted gene network
IVF	In vitro fertilization
KMT	Lysine methyltransferase
LncRNA	Long non-coding RNA
MBD1	Methyl-CpG-binding domain protein 1
MDR1	Multidrug resistance protein 1
MeCP2	Methyl-CpG binding protein 2
MEG3	Maternally-expressed 3
NAFLD	Nonalcoholic fatty liver disease
NASH	Nonalcoholic steatohepatitis
NTCP	Na^+^-taurocholate cotransporting polypeptide
P450	Cytochrome P450
PEG1	Paternally-expressed gene 1
PCAF	P300/CBP-associated factor
PKM2	Pyruvate kinase isozyme M2
PRC2	Polycomb repressive complex 2
SETDB1	SET domain bifurcated 1
SHP	Small heterodimer partner
SLC38A4	Solute carrier family 38 member 4
SUV39H1	Suppressor of variegation 3–9 homolog 1
TERT	Telomerase reverse transcriptase
VEGF	Vascular endothelial growth factor
ZEB1	Zinc finger E-box-binding homeobox 1
ZEB2	Zinc finger E-box-binding homeobox 2
ZHX2	Zinc fingers and homeoboxes 2
